# A fatal case of poisoning with a cathinone derivative: α-PiHP and its postmortem distribution in body fluids and organ tissues

**DOI:** 10.1093/jat/bkad026

**Published:** 2023-05-02

**Authors:** Paulina Wachholz, Rafał Celiński, Beata Bujak-Giżycka, Rafał Skowronek, Natalia Pawlas

**Affiliations:** Department of Pharmacology, Faculty of Medical Sciences in Zabrze, Medical University of Silesia in Katowice, 38 Jordana St., Zabrze 41-808, Poland; Toxicology Laboratory ToxLab, 6 Kossutha St., Katowice 40-844, Poland; Toxicology Laboratory ToxLab, 6 Kossutha St., Katowice 40-844, Poland; Department of Clinical Pharmacology, Faculty of Medicine, Jagiellonian University Medical College, 16 Grzegórzecka St., Kraków 31-531, Poland; Department of Forensic Medicine and Forensic Toxicology, Faculty of Medical Sciences in Katowice, Medical University of Silesia in Katowice, 18 Medyków St., Katowice 40-752, Poland; Department of Pharmacology, Faculty of Medical Sciences in Zabrze, Medical University of Silesia in Katowice, 38 Jordana St., Zabrze 41-808, Poland

## Abstract

New psychoactive substances continue to appear on the drug market, and alpha-pyrrolidinoisohexanophenone (α-PiHP) is one of the most popular cathinone derivatives. In this article, we report a case of death caused by α-PiHP. Based on the toxicological results of the studied case along with autopsy, histopathological findings and crime-scene information, fatal intoxication with α-PiHP was accepted as the final cause of death. α-PiHP and its metabolite (OH-α-PiHP) were detected and quantified in all postmortem materials (blood collected from the heart, the femoral vein and the dural venous sinuses; vitreous humor; cerebrospinal fluid; cerebral cortex; brainstem; cerebellum; bile; liver; kidney; heart; pancreas; spleen; thyroid gland; lung; adipose tissue; stomach and intestine). To date, this is the first case of determination of α-PiHP and its metabolite in postmortem specimens. In our opinion, α-PiHP and its metabolite concentration database can be helpful in the interpretation of fatal cases.

## Introduction

According to the Polish Sanitary Inspector in his warning on 11 July 2022 (1), for several months, there has been an alarming increase in the number of identifications of the dangerous substance α-pyrrolidinoisohexanophenone (α-PiHP) on the Polish market, also known as α-PHiP, 4-Me-PVP, 4ʹM-PVP, “Funky” and “A-PiHP”. This synthetic cathinone is classified as a forbidden substance in Poland. α-PiHP was first reported to the European Monitoring Centre for Drugs and Drug Addiction in Slovenia in December 2016 (2). The compound is an analogue of the psychotropic substances alpha-pyrrolidinopentiophenone (α-PVP) and alpha-pyrrolidinohexanophenone (α-PHP). Used alone and in combination with other substances, α-PiHP was the cause of serious poisoning and even death (3, 4). The reason for overdosing can be a lack of consumer awareness regarding the composition and concentration of the product because the effect of α-PiHP after oral administration appears following a long delay (30–60 min) when compared to that of α-PHP or α-PVP (2–20 min), which may create a risk of accidental overdose and death as a consequence (3). α-PiHP can be administered by oral, intravenous, rectal or nasal insufflation, inhalation (vaping) or inhalation after heating foil. This substance takes mainly the form of powder and crystals (5). After taking α-PiHP, a person may experience overstimulation hallucinations, delusions, psychoses, insomnia, anorexia and thirst, nausea, photophobia, trismus, heart problems (arrhythmias), tachycardia, vasoconstriction, high blood pressure, chest pain, breathing problems, shortness of breath and death (1, 3). The aim of this article is to present the first case of determination of α-PiHP and its metabolite in different postmortem specimens.

## Case History

A 37-year-old man (height 186 cm, athletic physique) was agitated and acting strangely, and then he lost consciousness. Witnesses called an ambulance, but after a resuscitative action, the doctor declared the man dead. The man was addicted to drugs. A bag of white powder labeled “α-PiHP” was found next to the corpse. The autopsy was performed by a forensic pathologist ∼48 h after death. The autopsy revealed cerebral edema and congestion, subconjunctival and subpleural petechial hemorrhages, pulmonary edema and congestion, low-grade atherosclerosis, liver enlargement, traces of rescue operations, fluidity and stagnation of blood in internal organs. The histological examination showed high-grade cerebral edema, brainstem edema, cerebellar edema and hyperemia, diffuse cardiac scarring, recent ischemic foci of myocardial fibers, cardiomyocyte hypertrophy, focal pulmonary edema and hyperemia, nodular goiter, hyperemia and chronic interstitial nephritis, chronic non-specific portal space inflammation in the liver, splenic congestion, pancreatic autolysis and fibrosis, increased congestion and colitis, erosive hemorrhagic gastritis and adrenal congestion. For toxicological analysis, samples of blood (collected from the heart, femoral vein and dural venous sinuses), vitreous humor, cerebrospinal fluid, cerebral cortex, brainstem, cerebellum, bile, liver, kidney, heart, pancreas, spleen, thyroid, lung, adipose tissue, stomach and intestine were collected during the autopsy. The collected samples were stored at −20°C until analyses, which were performed within 5 days from the autopsy.

## Experimental

### Certified reference materials and reagents

α-PiHP at a concentration of 1 mg/mL in methanol was acquired from Chiron (Trondheim, Norway). Mephedrone-d_3_ at a concentration of 0.1 mg/mL was chosen as the internal standard (IS) and was acquired from Sigma-Aldrich (Dorset, UK). All the remaining reagents used in the analyses were of high purity grade (high-performance liquid chromatography (HPLC) and mass spectrometry grade). Acetonitrile, 35% ammonia solution, ammonium formate, ethyl acetate, formic acid, methanol and water Chromasolv were acquired from Sigma-Aldrich (Saint Louis, MO, USA).

### Preparation of calibration samples

To quantify α-PiHP in the autopsy specimens, the standard addition method was employed. Mephedrone-d_3_ spiking solution was prepared for use as the IS to obtain a final concentration of 100 ng/mL in each body fluid sample and 100 ng/g in homogenates of each of the tissues. Together with the analyzed samples, calibration samples were prepared. Calibration samples were obtained through the addition of the appropriate volumes of the working solutions to 0.2 mL of each body fluid to obtain the final concentrations of 20, 50, 100, 200, 500, 1,000 and 5,000 ng/mL and 0.2 g of each homogenized tissue to obtain the final concentrations of 100, 1,000 and 5,000 ng/g (*n* = 3 for each level).

### Preparation of biological specimens

The liquid–liquid extraction procedure was used to prepare biological samples. To 0.2 mL of each body fluid containing mephedrone-d_3_ as the IS, including calibration samples, 15% ammonia (pH 12) and 0.5 mL acetonitrile were added. The mixtures were briefly vortexed and ultrasonicated for 60 min. Then, to each sample, 2.0 mL of ethyl acetate was added. Finally, the test tubes were shaken for 2 h and centrifuged, and the supernatant layer was collected and evaporated to dryness in a stream of nitrogen at ambient temperature. The dry residues were dissolved in 0.5 mL methanol and the samples were transferred to the glass inserts placed in the vials and they were ready for the analyses using liquid chromatography–mass spectrometry (LC–MS) and liquid chromatography–tandem mass spectrometry (LC–MS-MS).

A 0.2 g of each tissue containing mephedrone-d_3_ as the IS, including calibration samples, was minced and homogenized. The extraction procedure was the same as described earlier.

### Preparation of the powder found next to the corpse

A 10-mg sample of the white powder was dissolved in 1 mL of methanol, ultrasonicated for 10 min and then centrifuged. The resulting solution was further diluted 1:100 v/v with methanol. This diluted sample extract was transferred to the glass insert and vial and then analyzed using gas chromatography–mass spectrometry (GC–MS).

### Analytical methods

For the routine qualitative screening of α-PiHP in biological samples, the LC–MS screening method for illicit drugs and new psychoactive substances (NPSs) was used. Extracts were analyzed using HPLC ACCELA connected to a linear ion trap mass spectrometer Finnigan LTQ (Thermo Scientific, Waltham, MA, USA). Chromatographic separation was performed using a Hypersil GOLD C18 (4.6 mm × 100 mm, 3 μm) analytical column (Thermo Scientific, Waltham, MA, USA). The mobile phase was composed of Solvent A (0.02 M aqueous solution of formic acid and 0.05 M aqueous solution of ammonium formate) and Solvent B (10% Solvent A and 90% acetonitrile). The injection volume was 10 μL. The flow rate was 0.5 mL/min. A 40-min gradient was used, changing the composition as follows: 5% Solvent B (0.00–2.00 min) and 60% Solvent B (2.00–32.00 min), where it remained isocratic for 2.00 min. It was followed by re-equilibration for 6 min. Samples were ionized in the electrospray ionization (ESI) mode with positive ionization, and the ion monitoring range was *m*/*z* 50–500.

The confirmatory analysis of α-PiHP in biological samples was carried out using LC–MS-MS multiple reaction monitoring. Extracts were analyzed using ultra-high performance liquid chromatography Dionex UltiMate 3000 liquid chromatograph (Dionex, Sunnyvale, CA, USA) connected to a TSQ Endura mass spectrometer (Thermo Scientific, Waltham, MA, USA). The chromatograph was equipped with an RP-MS Accucore column (100 × 2.1 mm; Thermo Scientific). The mobile phase was composed of Solvent A (0.02 M aqueous solution of formic acid and 0.05 M aqueous solution of ammonium formate) and Solvent B (10% Solvent A and 90% acetonitrile). The injection volume was 10 μL. The flow rate was 0.5 mL/min. A 14-min gradient was used, changing the composition as follows: 10% Solvent B (0.00–0.50 min) and 95% Solvent B (0.50–7.00 min), where it remained isocratic for 1.00 min. It was followed by re-equilibration for 6 min. Samples were ionized in the ESI mode with positive ionization. The LC–MS-MS screening method had been validated for α-PiHP and OH-α-PiHP. Tandem MS conditions are presented in Table I.

**Table I.** LC–MS-MS Parameters Optimized for α-PiHP and OH-α-PiHP

Compound	Retention time (min)	Precursor (*m*/*z*)	Product (*m*/*z*)	Collision energy (v)
			91.055	22.6
α-PiHP	3.97	247.17	105.222	27.2
			140.220	24.9
			72.081	17.2
OH-α-PiHP	4.20	248.20	173.120	24.3
			230.190	27.7

For the GC–MS analysis of the white powder, the Thermo Trace 1310 gas chromatograph (Thermo Scientific, Waltham, MA, USA) coupled with the ISQ LT single quadrupole mass spectrometer (Thermo Scientific, Waltham, MA, USA) was used. The analyses were carried out with the use of the Rxi®-5Sil MS column (15 m × 0.25 mm, film thickness 0.2 µm; Restek, Bellefonte, PA, USA). The following working parameters were employed: injector temperature, 260°C; oven temperature, 100°C for 2 min, ramp at 20°C/min to 260°C; carrier gas (helium) flow rate, 1.2 mL/min; MS transfer line temperature, 250°C; MS source temperature, 250°C; and injection volume, 1 μL, on splitless mode. The obtained mass spectra in the positive electron impact ionization (EI) mode were compared with the spectra from the Cayman Chemical EI mass spectra library (2022) (Cayman Chemical).

The results obtained were processed with the use of the Xcalibur v. 4.0. program.

The validation data for the LC–MS method for α-PiHP are presented in the supplementary material.

## Results

**Figure 1.**
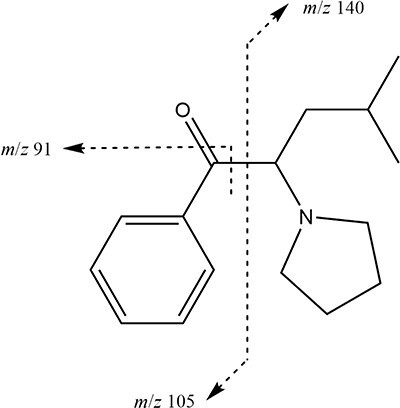
The fragmentation pathway of α-PiHP.

**Figure 2.**
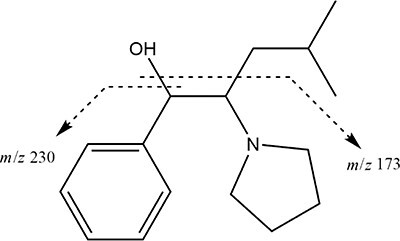
The fragmentation pathway of OH-α-PiHP.

As a result of the performed analyses, high concentrations of α-PiHP (*m*/*z* 246) were detected in all body fluids and tissues, which was responsible for the death of a man. The presence of α-PiHP metabolite (*m*/*z* 248) was also detected in all body fluids and tissues. The molecular formula of this metabolite corresponded to a reduction of the β-ketone moiety in α-PiHP to the corresponding alcohol metabolite. The product ion *m/z* 230 was obtained in the MS2 (tandem) mode from the precursor ion *m/z* 248, indicating the elimination of one water molecule from the protonated molecular ion [M + H − H_2_O]^+^ . This transformation is a well-known feature of the cathinones. The second product with *m*/*z* 173 suggested a water loss and a dealkylation. The proposed fragmentation pattern of α-PiHP and OH-α-PiHP is presented in Figures 1 and 2, respectively. The standard addition method was used to determine the concentrations of α-PiHP in each of the body fluids and solid tissues. The correlation coefficients for all calibration curves were >0.99. The extraction recoveries were >82.2%. Due to the lack of an OH-α-PiHP standard, concentrations of this metabolite were estimated based on the α-PiHP calibration curves. We expected a similar instrument response for drug and metabolite because of the similar chemical structure of both compounds. However, this is a significant limitation of this study, so the metabolite concentrations should be interpreted cautiously. The concentration ranges of α-PiHP and OH-α-PiHP in different specimens were tested at 9–7,352 ng/mL (ng/g) and 18–310 ng/mL (ng/g), respectively, as presented in Table II. Lidocaine was also detected in some materials. The analyses did not reveal any alcohol, classic drugs and other NPSs. α-PiHP was also identified in the white powder. The purity of α-PiHP was 91.3% (weight).

**Table II.** α-PiHP and OH-α-PiHP Concentrations (ng/mL and ng/g) and Their Ratios in Postmortem Specimens

Matrix	α-PiHP	OH-α-PiHP	α-PiHP/OH-α-PiHP
Femoral blood	2,377	223	10.66
Heart blood	1,133	28	40.46
Dural venous sinuses blood	966	26	37.15
Vitreous humor	1,492	58	25.72
Cerebrospinal fluid	1,350	34	39.71
Cerebral cortex	852	25	34.25
Brainstem	1,506	35	43.05
Cerebellum	1,016	25	40.34
Bile	540	187	2.89
Liver	504	310	1.63
Kidney	862	40	21.75
Heart	994	28	35.88
Pancreas	9	26	0.34
Spleen	2,146	48	45.01
Thyroid gland	1,463	38	38.57
Lung	1,991	51	38.98
Adipose tissue	14	21	0.67
Gastric/stomach	7,352	82	89.57
Intestine	602	18	33.71

## Discussion

**Figure 3.**
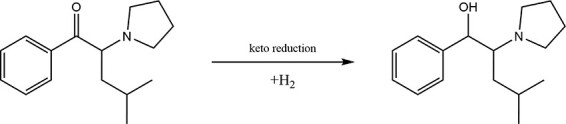
The metabolic pathway of α-PiHP.

To our knowledge, this is the first report to demonstrate the distribution of α-PiHP and its metabolite in body fluids and tissues in a real postmortem case. The major metabolic pathway of α-PiHP seems to be keto reduction since the only metabolite found was OH-α-PiHP (Figure 3), which confirmed the proposed model (6). The same pathway of metabolism was described for the analogue compound α-PHP (7, 8). In the present case, the femoral blood concentration of α-PiHP was 2,377 ng/mL, which is much higher than those previously reported (3, 4). Relative to histological analyses, whose findings revealed high-grade cerebral edema, brainstem edema, cerebellar edema and hyperemia and focal pulmonary edema and hyperemia, it clearly explains the rapid reaction of the man’s organs and his eventual sudden death. During the study, we focused further on the distribution of α-PiHP in the different specimens that we analyzed. As shown in Table I, we observed that the concentrations of α-PiHP measured in the heart and femoral blood significantly differ, with concentrations of 1,133 and 2,377 ng/mL, respectively. In forensic toxicology, postmortem redistribution (PMR) is defined as the ratio of xenobiotic concentration in blood from the heart to peripheral blood from the femoral vein (*C*/*P*). However, in some circumstances, using the ratio of xenobiotic concentration in the liver to the peripheral blood (L/P) or both factors is more accurate. New redistribution assessment coefficients are being sought. When the *C*/*P* ratio is <1.0 (in the present case, this ratio is ∼0.5), the drug is not prone to redistribution, but resuscitation attempts may result in a *C*/*P* ratio <1.0 (9, 10). The liver *L*/*P* ratio has been recently proposed as a marker for PMR, with ratios >20 indicative of a propensity for significant PMR and ratios <5 indicating no propensity toward PMR (9). In the present case, this ratio is ∼0.2, which also shows a lack of PMR. Previous studies have shown that pyrrolidines, such as α-PVP, appear to be less susceptible to PMR. α-PiHP has a similar structure to α-PVP, and our results may confirm its low redistribution (11). The heart blood α-PiHP concentration (1,133 ng/mL) was in good agreement with that in vitreous humor (1,492 ng/mL) and cerebrospinal fluid (1,350 ng/mL). In previously published studies, the distribution of other cathinone derivatives in vitreous humor relative to the blood has been highly variable (11). The high concentration in stomach contents strongly suggests that the man took α-PiHP orally. In humans, the analogue compound α-PHP is mainly eliminated in the urine in unaltered and metabolized forms. α-PHP is mainly metabolized in the liver to form OH-α-PHP and others (8). In the present case, the estimated concentrations of OH-α-PiHP were significantly lower than those of α-PiHP, which suggests acute poisoning and sudden death. The parent drug-to-metabolite ratio has been calculated for all the fluids and tissues. Table II presents the α-PiHP/OH-α-PiHP ratios. The concentration of α-PiHP in the stomach was found to be 7,352 ng/g representing the most abundant compound with the highest α-PiHP/OH-α-PiHP ratio of 89.57. As shown in Table II, the concentrations of α-PiHP were much higher than those of its metabolite, except for the pancreas and adipose tissue with an α-PiHP/OH-α-PiHP ratio of 0.34 and 0.67, respectively. The α-PiHP and its metabolite concentrations in the liver and the adipose tissue were the most similar with an α-PiHP/OH-α-PiHP ratio of 1.63 and 0.67, respectively. However, the concentrations in the liver were significantly higher than in the adipose tissue. OH-α-PiHP PMR seemed to be lower than α-PiHP PMR, so the OH-α-PiHP migration rate between tissues and blood is low. α-PHP and another similar cathinone derivative 3,4-methylendioxypyrovalerone seem to have high blood–brain barrier (BBB) permeability (12–14). In the present case, high concentrations of α-PiHP in different parts of the brain can confirm that α-PiHP easily crosses the BBB, and this may be the reason why α-PiHP is currently popularly abused.

## Conclusions

This article presents the identification and quantification of the cathinone derivative α-PiHP and its metabolite (OH-α-PiHP) in postmortem human specimens. This is the first study that presented the distribution of α-PiHP and its metabolite OH-α-PiHP in postmortem biological fluids and tissues from cases. The concentrations of metabolites combined with the parent drug concentration can be helpful in the interpretation of fatal cases involving α-PiHP and contribute knowledge to the scarce data reported in the international literature. The detection of relevant metabolites in addition to the parent drug may be useful for other researchers and scientists working in the field of clinical and forensic toxicology. The presence of α-PiHP metabolite in biological specimens can be crucial for indisputably proving the ingestion of the drug, so the abuse or intoxication with α-PiHP may be provable unequivocally. The presented case also shows that the evaluation of toxicological results along with histopathological alterations and circumstantial information is fundamental in determining the correct cause of death.

## Supplementary Material

bkad026_Supp

## Data Availability

The data underlying this article is available in the article.
