# Supplementary material for: A fatal case of poisoning with a cathinone derivative: α-PiHP and its postmortem distribution in body fluids and organ tissues
Source: J Anal Toxicol. 2023 May 2;47(6):547–51. doi: 10.1093/jat/bkad026 (PMC10362950; doi:10.1093/jat/bkad026)
Supplement: bkad026_Supp [file bkad026_supp.zip › jat-23-3937-File008.docx]

The HPLC α-PiHP quantification method was validated. The following attributes were assessed during validation: linearity, including the limit of detection (LOD), the limit of quantification (LOQ), intraday and interday repeatability of target compound for each matrix, matrix effects and recoveries for each type of biological specimens.

Linearity was evaluated by making five calibration curves on five days. To quantify α-PiHP within the autopsy specimens, the standard addition method was employed. Mephedrone-D3 spiking solution was prepared for use as the IS to obtain a final concentration of 100 ng/mL in each body fluid sample and 100 ng/g in homogenates of each of the tissues. Together with the analysed samples, calibration samples were prepared. Calibration samples were obtained through the addition of the appropriate volumes of the working solutions to 0.2 mL of each body fluid to obtain the final concentrations of 20, 50, 100, 200, 500, 1000 and 5000 ng/mL and 0.2 g each homogenized tissue to obtain the final concentrations of 100, 1000 and 5000 ng/g (n = 3 for each level), then extracted in accordance to the sample preparation step. The seven-point calibration curves prepared for body fluids standards and the three-point calibration curves prepared for tissues were in the linear range of 10–5000 ng/mL and 100-5000 ng/mL respectively, with coefficients greater than 0.99. The limit of detection [LOD, signal-to-noise (S/N) ratio = 3] and limit of quantification [LOQ, (S/N) ratio = 10] were calculated. The LOD and LOQ values were 3 and 10 ng/mL for body fluids and 5 and 15 ng/g for solid tissues, respectively. We had no blank human specimens, so it was not possible to present the usual accuracy and precision. However, we evaluated the intraday and interday repeatability of the target compound for each biological specimen. The results are shown in Table I.

**Table I** Intraday and interday repeatability for determination of α-PiHP in each human specimen

| Specimen | Repeatability (% RSD) | |
| --- | --- | --- |
|  | Intraday (n = 5) | Interday (n = 5) |
| femoral blood | 15 | 5.8 |
| heart blood | 10 | 8.2 |
| dural venous sinuses blood | 10 | 11 |
| vitreous humour | 13 | 7.6 |
| cerebrospinal fluid | 7.1 | 5.5 |
| cerebral cortex | 14 | 12 |
| brainstem | 14 | 15 |
| cerebellum | 20 | 16 |
| bile | 14 | 10 |
| liver | 6.2 | 11 |
| kidney | 17 | 15 |
| heart | 8.5 | 7.4 |
| pancreas | 27 | 15 |
| spleen | 18 | 11 |
| thyroid gland | 16 | 14 |
| lung | 13 | 9.9 |
| adipose tissue | 10 | 7.2 |
| gastric/stomach | 24 | 22 |
| intestine | 17 | 13 |

To determine matrix effects and recovery rates in all biological specimens, three types of solutions were analyzed. The matrix effect and recovery rate were calculated as follows. Matrix effect (%) = [(A − B)/C] × 100. Recovery rate (%) = [B/(A − B)] × 100. The first solution described as A was obtained after reconstitution with methanol containing the 100% recovery concentration of the target compound for the same sample extract (through peak area). The second solution described as B was obtained from a matrix after reconstitution of the sample extract residue with pure methanol (through peak area). The last solution described as C was a methanol solution without any reconstitution, but only with the above 100% recovery concentration of the target compound (neat sample). The matrix effects and recoveries for each type of biological specimen are shown in Table II.

**Table II** Matrix effects and recoveries for each type of biological specimens

| Specimen | Matrix effect ± SD (%) | Recovery ± SD (%) |
| --- | --- | --- |
| femoral blood | 85.1±6.1 | 91.1±8.0 |
| heart blood | 74.9±9.0 | 92.1±8.5 |
| dural venous sinuses blood | 79.1±3.0 | 91.9±6.4 |
| vitreous humour | 77.7±3.6 | 86.1±0.3 |
| cerebrospinal fluid | 79.7±7.6 | 90.7±1.2 |
| cerebral cortex | 70.2±6.0 | 83.3±5.9 |
| brainstem | 74.3±10 | 84.8±1.9 |
| cerebellum | 78.1±8.7 | 91.2±1.7 |
| bile | 77.3±5.4 | 83.6±7.8 |
| liver | 73.4 ±0.8 | 84.1±3.8 |
| kidney | 77.1±8.5 | 89.9±3.6 |
| heart | 78.2±6.9 | 85.2±0.8 |
| pancreas | 79.6±3.2 | 87.4±1.1 |
| spleen | 70.5±5.2 | 83.7±5.2 |
| thyroid gland | 69.7±0.5 | 91.6±8.2 |
| lung | 73.2±2.5 | 86.2±5.8 |
| adipose tissue | 79.6±8.6 | 84.3±3.9 |
| gastric/stomach | 69.5±2.0 | 82.2±8.8 |
| intestine | 75.7±6.6 | 89.4±2.4 |

Data given as mean ± SD (n = 3)
